# Housing coalition dynamics: a comparative perspective

**DOI:** 10.1057/s41295-021-00245-6

**Published:** 2021-05-10

**Authors:** Valesca Lima

**Affiliations:** Department of Sociology, Maynooth University Social Sciences Institute (MUSSI), Maynooth University, Iontas Building, North Campus, Maynooth Co Kildare, W23 F2H6 Ireland

**Keywords:** Coalitions, Alliance building, Housing, Social Movements Outcomes, Consequences

## Abstract

Social movement coalitions are a vital component within the dynamics of political mobilization. While previous research has established why and how coalitions emerge and dissolve, how they are maintained and the outcomes they generate have been less explored, especially in housing studies. This research contributes to the study of movement coalitions through an empirical examination of the dynamics of how coalitions interact, cooperate, and sustain alliances, in addition to exploring the outcomes that are produced as a result of these coalitions. It draws upon a comparative approach of housing coalitions in Dublin and Lisbon, where local housing groups have played a critical role in protesting against housing injustices and in articulating alternative policy solutions to the housing crisis. In mapping the diversity of coalition members, this research finds that tolerance for difference and negotiation capacity impacts how long coalitions last as well as the outcomes that they lead to. This study contributes to the study of coalitions by analyzing the relationship between outcomes and the mechanisms which sustain coalitions using a comparative framework.

## Introduction

More than a decade has passed since the property bubble burst, but many capital cities have been saddled with a worsening housing crisis, where homeownership rates have dropped, evictions and homelessness have increased sharply, and affordable accommodation is scarce. When hundreds of people gathered at the Largo do Intendente, a historical square in Lisbon’s city center, on September 22, 2018, to demand more affordable housing and to protest against real estate speculation, they wanted to call attention to housing precarity. This precarity exists as a result of the inability of the markets and housing system to respond to housing needs. Called by a broad and diverse alliance of entities, “Morar em Lisboa” (Living in Lisbon), the protesters arrived with posters and banners to call for the right to a decent home. Days later, on October 3, 2018, a similar event took place but this time in Dublin city center. Organized by the “Raise The Roof” campaign-style coalition, this diverse group was calling for the government to declare the housing and homeless crisis an emergency, demanding more investment in public housing and rent controls. The two protest events were geographically far apart but close in their origins and goals: the event in Lisbon was led by a group of somewhat loosely organized coalition of entities campaigning for housing rights; the Dublin one was organized by a more structured coalition, but the objectives were very similar. Both groups have the right to housing at the center of their demands. They are part of an increasing number of movements concerned with housing issues and social justice, coming together to demand major political action on the housing crisis. While diverse in their composition, experience and strategies, housing coalitions have become an important site of mobilization for housing rights.

There is an extensive body of literature that recognizes the importance of coalitions as a critical element within the dynamics of social movements. Social movement scholars have pointed to the complex nature of these movements and highlighted the relationships forged among groups when orchestrating joint political action (McCammon and Moon 2015; Heaney and Rojas 2008; McCammon and Campbell 2002). These studies have shown the importance of taking advantage of political opportunities when building strategies in order to increase the chance of success, indicating that coalition building is a commonplace strategy used by social movement organizations to achieve their goals (van Dyke and Amos 2017; Meyer and Corrigall-Brown 2005; Staggenborg 1986).

I develop these ideas in an analysis of contemporary housing movement coalitions in Dublin (Raise The Roof—RTR) and Lisbon (Morar em Lisboa—MEL) using a comparative approach. More specifically, this article asks the following question: what are the constitutive and longevity aspects of housing coalitions and how do those aspects influence outcomes? Research in the field has advanced our understanding of the factors that encourage coalition formation: the structures of political opportunities and threats, the existence of previous ties between coalition members, and congruent ideology and resources (Soule, 2013). The next step is to build on the processes through which movements sustain coalitions as well as the outcomes of such collaborations, in particular, with empirical comparative research on the formation and outcomes of movement coalitions in the field of housing studies. This is the research gap I attempt to focus on in this study.

Capturing the lack of conceptual clarity in coalition studies, I use the terms coalitions and alliances interchangeably, following Staggenborg (2015a) and van Dyke and McCammon (2010). The use of terms such as “networks,” “alliances,” “collectives,” “movement” and “platforms” became common among activists to describe both formal and loosely connected groups of activists. While networks have a more complex significance and might have different purposes (see Tarrow, 2005), some activists feel that the term “coalition” is too hierarchical and exclusionary (Staggenborg 2015a). It might be the case that some coalition members understand coalitions as organizational alliances rather than looser collections of unaffiliated individuals and informal organizational representatives (Staggenborg, 2015b).

I begin with a discussion on the literature regarding social movements in coalitions, with particular attention on the effects of coalitions on the movements, the factors that lead to coalition formation, as well as how alliances are maintained. This discussion is followed by a presentation of the research methodology. Attention is then turned to mapping coalitions and the dynamic nature of housing movement coalitions in the respective cities, as I analyze and discuss the findings. In the discussion and conclusion, I reflect on the research findings and contributions while also suggesting areas for further research.

## Theoretical and conceptual frameworks in the field of movement coalitions

Social movement coalitions materialize when distinct activist groups enter into an agreement to cooperate and collaborate towards common goals. Coalitions are interorganizational agreements formed for the purpose of collectively addressing a specific set of policy or political objectives (Heaney and Rojas 2008), involving cooperative efforts that may include a “single project” or “multiple activities over time” (van Dyke and McCammon 2010). Over the years, research investigating coalitions has focused on how and why coalitions form and the challenges involved in building these alliances. This research has emphasized the increasing use of coalitions as tools to settle differences among activists and organizations (Heaney and Rojas 2008; Bandy and Smith 2004; van Dyke and McCammon 2010). Coalitions are clearly important when it comes to social change: when activists and organizations join forces to act toward common goals, they are likely to be more successful in achieving those goals than if various individuals and groups act alone (Staggenborg, 2015a). Focusing on why, how, and under what circumstances activists choose to cooperate highlights central issues at the heart of understanding contemporary social movements (Meyer and Corrigal-Brown 2005, p. 328).

Social movements are not necessarily equivalent to coalitions, but they represent one of their building blocks (Tarrow 2005, p. 164), with some authors referring to social movements as “nested coalitions” (Della Porta and Diani 2015). Before joining a coalition, organizations consider the costs and benefits of collaboration, including time spent in meetings, effects on the organization’s identity and public image, and the risk of sharing or withholding information (Moreno-García and Torres-Martínez 2020; Wells et al. 2009; Valocchi 2009). The running of a coalition is difficult work. Coalitions often sow together the movement’s moderate and radical strands, which are regularly in conflict with one another (Heaney and Rojas 2008). According to van Dyke and Amos (2017, p. 9), it is difficult to separate coalition outcomes from general social movement outcomes, due to the movements and coalitions having the same goals, to a certain extent. The literature identifies four different coalition outcomes: survival, organizational change, movement mobilization, and political outcomes. The relationship between coalition dynamics and outcomes has received less attention from this specialized literature. In this study, I examine coalition outcomes in terms of “success” or “effectiveness” when it comes to political outcomes, as I consider the extent the movements were able to organize joint demonstrations, gain mass media attention, pool resources and coordinate efforts—all of these are signs of a successful or effective coalition (Staggenborg 2015b).

The level of cooperation among members varies significantly from coalition to coalition and among groups within the coalition, since some groups are at the core of planning and actions while other groups have a more marginal role (Meyer and Corrigal-Brown 2005; Hula 1999). As noted by Tarrow (2005, p.167), the extent of cooperation varies from the point of view of at least two dimensions, level of commitment and time. Cooperation encompasses the endorsement of some actions and claims of groups in the coalition and this can range from adding a group’s name to a manifesto or website to coordinating strategy, dividing tasks, pooling resources, all the way up to forming a permanent umbrella organization. The second dimension of cooperation is a temporal one. Groups can maintain “event coalitions,” which are temporary alliances created to organize actions around specific protests or events (Levi and Murphy 2006), or they can make permanent arrangements for a permanent collaboration. In this case, a coalition becomes a distinct organization with hired staff, membership, and funds to organize events—and social movements can even turn into NGOs (Levi and Murphy, 2006; Tarrow, 2005). The degree of formalization and permanency varies according to the intensity of the ties between the activists. Di Gregorio (2012) found that some alliances are structured around strong bonds with frequent interactions among participants, while others have few concrete exchanges. Studies by van Dyke (2003) and Mix (2011) suggest that certain coalitions are shaped around single issues (e.g. reproduction rights), while others are more expansive and include multiple issues (e.g. gender equality, sexual reproductive health, women's political participation).

Various studies have explored the circumstances facilitating and obstructing coalition formation (Soule 2013; van Dyke and McCammon 2010; Levi and Murphy 2006; Staggenborg 1986, 2015a; Almeida 2010; Tarrow 2005). In light of these studies, it is possible to identify in the literature four prominent factors critical to coalition formation. These are: political context, social ties, shared ideology and available resources. Previous studies have established that the presence of strong social ties is conducive to groups participating in coalitions (Heaney and Rojas 2014; Rose 2000; Reese et al. 2010). Interpersonal social ties across groups are reflected in the level of interaction, which can profoundly shape how coalitions are formed and what they look like (van Dyke and Amos 2017, p.4). Whether a coalition forms or not is closely related to the presence of “bridge builders” or “coalition brokers.” These are individuals with multiple affiliations across varied groups who are able to foster connections between movements, even those that are not in the same area (Soule 2013; Rose 2000). In her study of coalition building in the anti-death-penalty movement, Jones (2010) shows that coalition projects focused on bridge-building between different grassroots anti-death-penalty communities, creating an effective model of advocacy on behalf of persons facing the death penalty. Another example is the study by Tomazini et al. (2016) focusing on the alliance between social movements and institutional actors engaged in advocacy for food security, in which collaboration was only possible due to the participants’ strong and diverse ties with progressive parties.

Several studies have indicated that a shared ideological orientation (Enriquez 2014; Cullen 2005; van Dyke 2003), cultural similarities between movements (Jung et al. 2014; Bandy and Smith 2004) and similar identities (Corrigall-Brown and Meyer 2010; Heaney and Rojas 2014) aid coalition formation. As suggested by van Dyke and McCammon (2010), ideological congruence is a critical condition that, in some cases, is sufficient to inspire a coalition. Research has also investigated the inverse: different ideologies hinder coalition formation (Polanska and Piotrowski 2015; Tarrow and Meyer 2018). However, it has been shown that common ideological grounds may not suffice as a condition for a coalition to work. A number of studies have shown that even when organizations share common goals and ideologies, and are also aware of one another, they still do not engage in collaboration (Soule 2013). Nevertheless, some organizations are able to find a common ground. As an example, the *Stop Climate Chaos* coalition, in which members of some of the organizations do not embrace climate justice, has built upon the work of the Working Group on Climate Change. Based on their shared understanding of the range of causes and implications of climate change in the Global South, participants in the coalition were able to create a common ground around the various groups (Saunders 2008).

Political threats are powerful motivators when it comes to collective action and one of the most powerful incentives for coalition formation (van Dyke and Amos 2017). As already established by political opportunity theory, the factors that are conducive to uniting the efforts of distinct groups of actors usually combine threats (Almeida 2010; Juskas and Edwards 2005; Tarrow 2005) and opportunities and also the availability of resources that organizations lack (Heaney and Rojas 2014).

As shown above, it is now well established from a wide range of studies that those factors (ideology, interactions, political context) influence the formation and success of coalitions. Taken together, these studies outline a critical role for research focused on the dynamics of coalition formation, as they allow us to see what elements need to be present for a coalition to succeed. However, they do little in terms of explaining other features that impact coalition maintenance and outcomes. I build on this previous framework to explore other emergent elements, such as cooperation, respectful interaction, the connecting of actors, and outcomes as the next step forward towards a better understanding of coalition longevity and outcomes that have been less discussed in the literature.

## Research design

A comparative approach was chosen for this research for two reasons. First, it allowed for a cross-national investigation of the factors influencing coalition maintenance and longevity. Second, the comparative element enables a narrative that provides ‘concrete knowledge about specific processes’ (Della Porta 2012) from specific country contexts. Besides epitomizing the current trend of housing unaffordability in Europe following years of economic austerity, Dublin and Lisbon are two European capital cities where there are energetic housing movements. These movements have attracted plenty of attention to housing issues, such as the increase in homelessness, gentrification, and skyrocketing rents.

The empirical analysis is based on interviews with housing activists, field observation of meetings and events, and documents produced by activists posted on websites and social media. The interviews were semi-structured, allowing for themes to emerge while following a standard interview script. All 45 interviews were recorded with the consent of the participants and anonymized during the data analysis. Interviews lasted between 50 min and one hour and a half. They took place in neutral locations between January and June 2019. Participants were chosen using a non-random approach based on the respondent’s involvement in housing mobilization. The sample included housing group members, elected politicians, homelessness NGO representatives, and activists. During the interviews, participants talked about their experiences in the housing movement, the coalitions they participate in, and their perception of success and challenges in housing mobilization. Interviews were transcribed and coded using NVivo, a qualitative software. Later, code and sub-codes were analyzed using a thematic analysis approach (Saldaña 2009). A descriptive element was adopted to explain the dynamics of each coalition (see Wendt 1998).

This research draws on data collected from two housing coalitions formed to address the housing crisis: Raise the Roof (RTR) in Dublin and Morar Em Lisboa (Living in Lisbon—MEL) in Lisbon. These groups were selected based on their housing rights activism and the public profile they have gained in recent years, but other smaller, less prominent coalitions do exist. Selecting these two groups meant that the study was empirically manageable but still politically varied. Raise the Roof (RTR) organize themselves more vertically and are a formal coalition. Morar Em Lisboa (MEL) is more horizontal with a particular structure (it is run by a committee, but it does not have a chair or a president). Most interviewees were people between 25 and 50 years old, many of them long time activists with extensive experience in community organization and housing. Secondary data, such as policy documents, open letters, manifestos and mission statements were collected from the coalitions’ social media, blogs and websites. I describe these groups in more detail in the next sections. As the data collection and interviews took place in the first half of 2019, I did not have an opportunity to analyze some of the housing developments that have taken place in both cities during the Covid-19 pandemic, such as rent freezes and mortgage moratoriums implemented in both Ireland and Portugal.

## Mapping housing coalitions

The recent trends of commodification, financialization, gentrification and hyper-tourism have become more apparent since the 2008 economic crash, leading to protests against austerity in Ireland and Portugal (Naugton 2015; Fernandes 2016). The many contradictions and tensions in the field of housing influenced the form that movements took following the crash. Housing inequality became a fertile ground for a new generation of social and urban movements, locally based and but also interconnected (Hearne et al. 2018; Seixas et al. 2019). In Dublin, housing mobilization went through two waves after the economic crash. The first wave (2008–2014) was a community-led mobilization against the course of urban regeneration during the boom of the Celtic Tiger years. The second wave (2014–present) refers to movements that emerged as the initial period of the crisis gave way to a new housing crisis, involving the lack of affordable homes in the private sector and increasing homelessness (Hearne et al. 2018). In Lisbon, and in Portugal in general, housing issues were not in the spotlight during the years of economic austerity—but this changed during the ‘recovery” period, with the appearance and consolidation of a number of activist groups and platforms from 2017 concerned with the right to housing (Tulumello, 2019). In both cities, the RTR and MEL coalitions have played a pivotal role in bringing together a wide coalition of actors fighting for the right to housing, amid newfound prospects for collective mobilization.

Created in October 2018, Raise The Roof (RTR) is an alliance-type campaign coalition of approximately 50 organizations that is engaged in a series of activities such as rallies, advocacy and lobbying, supported by homelessness NGOs, leftist parties, student unions, trade unions, and local housing groups, a novel arrangement in the contemporary landscape of housing mobilization in Ireland. While this coalition has housing groups, it is also made up of entities not directly related to housing, such as trade unions and women’s groups. Even if organizations such those being analyzed are considered uncharacteristic of housing coalitions, the political context of the housing and homeless crisis has affected their members, providing a favorable situation for mobilization and coalition formation. The RTR campaign, coordinated by the Congress of Trade Unions (ICTU), assembled a wide range of groups engaged with housing issues, appealing to the wider public with a well-defined narrative for the housing crisis and clear solutions for it. The ICTU presented its own Charter for Housing Rights, encompassing its key principles for solving the housing crisis. Speaking about the efforts to mobilize more people, one participant from a women’s organization explained that “the outlook of all the organizations involved now was: okay, how do we make this wider?” (Participant 11). The efforts and resources mobilized led to one of the biggest housing and homeless demonstrations in Ireland. The RTR rally on October 3, 2018, had a large turnout with 10,000 people in attendance. Another demonstration in May 2019 was attended by nearly 15,000 people. Other factors also contributed to those large turnouts such as the presence of students and the support of other smaller coalitions, such as Take Back The City and the Irish Housing Network, which also supported the protests. These coalitions emerged in order to bring together housing activism efforts in Dublin and Lisbon, and a summary of these coalitions is presented in Table 1.

**Table 1:** Coalitions

Coalition	Members	Mission Statement	Types of Claims	Ideological Alignment
Raise The Roof (RTR) (2018)	Approximately 50 members (trade unions, housing agencies, women’s groups, political parties, advocacy groups, community organizations, student unions, housing academics)	Raise the Roof has demanded a radical shift in housing policy, founded primarily on the restoration of the State’s central role in housing provision	The right to housing, building social housing, introduction of rent control, secure tenancies, end homelessness	Liberal, progressive, and leftist participants
Morar em Lisboa (MEL) (2017)	Over 50 associations + approx. 5,000 individual signatories to a letter Neighborhood associations, trade unions, tenant unions, NGOs, housing activists, academics	MEL is an open forum composed of citizens who want to see Lisbon's housing policies being discussed and urgently changed	End evictions, resettlement of evicted families, put vacant buildings to use, regulation of short-term letting, against gentrification and rent control	Liberal, progressive, and leftist participants

The movement Morar em Lisboa (MEL) was created in 2017, making its first public appearance in January with an open letter denouncing the tourism boom in the city of Lisbon and asking for a “new housing and spatial planning policy” as well as alerting authorities to the fact that living in the capital is today a “right practically inaccessible to Portuguese families.”^1^ The letter received a good public response and media coverage, gaining thousands of signatories in the months that followed. MEL advocates for the right to housing and to the city and is at the forefront of the national political debate in Lisbon, while focusing also on drawing the attention of civil society to housing-related issues (Mendes 2020). The open letter turned out to be a strong point of connection among activists around the housing problem, leading to the formation of groups that had already existed to a certain extent but were too dispersed. Together, the groups and individuals that signed the letter created a new way to communicate with the public, as MEL is focused on organizational flexibility, public discussion and horizontality of representation. Commenting on the expansion of the group, one of MEL’s first signatories said “MEL was gaining space in public opinion because it has great media impact. We work a lot also through Facebook, online, so after a while we started to gain some representation with politicians” (Participant 39). The group grew to be an important forum for dialogues with various political and institutional actors responsible for housing in the city, being invited for parliamentary hearings and to media appearances. MEL is coordinated by a committee of neighborhood associations and strongly supported by academics.

The emergence and participation of so many housing groups suggests a keen interest in a sustained housing movement in Ireland, even if the housing activists have not yet been able to create an inclusive and sustained housing movement. The complex and diverse housing groups and coalitions that emerged in Lisbon in response to housing financialization and gentrification have been able to highlight the struggles of the urban poor. But how do members of MEL and RTR navigate in this complex, multi-actor, inter-organizational space to cooperate and sustain their coalitions? I examine these questions next.

## Cooperation, conflict and outcomes in housing coalitions

### Creating alliances and coalition building

RTR involves experienced and resourceful activists, such as trade union members and political parties, who have aided the coalition in reaching the wider public. After a successful conference organized by the ICTU, the diverse groups in attendance agreed on the financial and unfair aspects of the housing crisis and observed that some smaller actions were taking place at local level. The logical conclusion then was to connect those groups around the same platform, as explained by this member of the RTR coalition, a representative of a trade union:

“We had a whole range of smaller campaigns who had been essentially saying more or less the same thing over a longer period of time, not necessarily connecting up. So, on foot of that meeting we agreed we should work together as best as possible and try to create a broader civil society platform” (Participant 14).

Morar em Lisboa (MEL) started off as a small collective of six people publishing an open letter in the Trienal de Arquitectura de Lisboa (Lisbon Architecture Triennale). Put together by academics, housing activists and other entities, MEL quickly expanded to connect people and groups against gentrification and for the right to housing and the city. The founders intended to connect the various groups engaged in similar activities and focused on denouncing the fast-paced gentrification Lisbon was going through and its impact on local residents. As reported by an activist who was one of the first signatories of the open letter:

We wanted a movement where we could denounce what is happening in this city. The issue of gentrification, a question that nobody was talking about at the time. The question of people being evicted when the rental contracts ended. It is a movement of denunciation and pressure and there were a lot of people thinking just that, so we brought them together (Participant 38).

Both the RTR and MEL coalitions intended to make the best use of their capacity to attract a variety of organizations and individual participants to secure the widest possible support for the goals of the coalition. When it comes to coalition formation, timing was also an important factor. While the political threat of the worsening housing crisis is likely to have acted as a powerful connection among actors and spurred cooperation among them, another two specific political opportunities contributed to the emergence of these coalitions in that specific time and context: the prospect of new legislation in Ireland and the approaching local elections in Portugal.

RTR organized a protest rally on October 3, 2018, to coincide with a vote on a multi-party motion in the Oireachtas (National Parliament) calling on the government to declare a housing emergency, a referendum on the right to housing, the building of more social housing and the provision of rent controls, among other demands.^2^ Considering that groups join coalitions when they see their efforts on a particular set of issues as urgent and potentially efficacious (Heaney and Rojas 2014; Meyer and Corrigall-Brown 2005), the multi-party motion spurred cooperation. If successfully passed, it was a concrete outcome and an important victory for the coalition. In fact, the public pressure created by the rally, and the direct attention on the motion, was successful and the motion was passed by a majority. The proposed bill was introduced by opposition parties: Sinn Féin, the Green Party, the Labour Party, People Before Profit, Solidarity, Independents4Change, and the Social Democrats (all members of RTR). Whereas the passing of the motion indicated the strong political clout built by RTR, the related legislation was defeated, with 36 members voting in favor and 60 voting against it.

A similar window of opportunity contributed to the formation of the MEL coalition. With the approaching Portuguese local elections in October 2017, the group understood it was a pertinent moment to pressure central and local governments to urgently act on the housing problem, taking the forthcoming election as an opportunity to sensitize civil society on the housing issue (Mendes, 2020). The interviewees explained that, up to that point, housing was not an important theme on the local political agenda—despite the increasing problem of displacement, evictions and rising rents—and housing interventions were scattered among different ministerial cabinets.^3^ This point is illustrated further by this housing activist:

At the time [local elections] we decided that it was a good time to form this movement because there was no government policy on housing. Lisbon Municipal Council did not have a program for low-income [earners], those people were being expelled from their homes. So, before the election MEL began to denounce this (Participant 40).

The symbolic force of both coalitions cannot be explained only by the quality of the arguments or by the worsening of the housing crisis because other initiatives, such as policy papers and op-eds had already been produced by activists in previous years. The political force of MEL and RTR seems to be their capacity to connect a wide range of political actors around a common problematic situation, producing an interorganizational environment where participants bridge-build and bond through their efforts to create a unifying message, at least for a period of time. As noted by Moura (2019), this type of approach employs bonds of solidarity and belonging, producing new opportunities for engagement and participation in collective action. As shown in Table 1, RTR and MEL are composed of diverse entities, indicating the breadth of the housing movement. Some of the coalition members do not have housing as their primary identity, but the coalitions were capable of mobilizing groups that had previously embraced other issues, making clear demands that focus on the role of the state in responding effectively to the housing crisis. This unifying master frame (Benford and Snow 2000) was a determinant for coalition building and the development of alliances.

### Sustaining coalitions

The broad membership of RTR and MEL is their greatest advantage, but it is also their biggest vulnerability. As noted by Benford (1993), differences among coalition groups are a weakness of social movements. The capacity to manage tensions within the coalition is a significant factor affecting the growth and maintenance of coalitions. While there is a clear benefit for coalitions when a range of participating organizations unify their capacities to pursue shared targets, real coalition work among different groups is difficult to realize. Ideological and strategic conflicts occur within RTR and MEL. Although the unified agenda of the various movements involves a rejection of the financialization of housing and a call for more state intervention in the provision of homes, their specific agendas, strategies and political views sometimes differ, even if some of their key ideologies overlap.

The first main difficulty in sustaining housing coalitions in Dublin is related to housing policy itself. While some coalition members demand a stronger role for the state in the provision of housing, less radical members believe that the private sector can provide affordable housing below market prices. This is the source of many debates among coalition members. A second point of dissent often takes place around the organization of marches. Some members are keen to organize people and take to the streets, but other members, such as trade unions, are more careful. To them, a poorly attended demonstration is a political loss and could jeopardize the legitimacy of the movement, as illustrated by this representative of a trade union, a member of RTR:

Marching is a public demonstration of legitimacy and it is a public demonstration of credibility and it is a number's game. If you announce that this is the greatest crisis in the history of the country, and you can only muster five people, well, then you are going to get commentators in the media that will say ‘no, it is not quite obviously a serious issue’ and move on. And the next time you say it, you will be ignored (Participant 26).

Large entities are very careful with their public image, so trade unions are more cautious with the decisions they make. The third difficulty mentioned by research participants is more tactical, relating to the challenge of assembling a wide range of groups and negotiating a plan of action. While some groups are more focused on lobbying and policy solutions (i.e. parties, NGOs and trade unions), smaller groups are also interested in community organizing and in constructing an inclusive housing movement. So, while some groups prefer to focus on institutional activities, others prefer to organize tenants and push for more direct action, especially housing collectives that employ a direct action approach, such as the Irish Housing Network (IHN) and Take Back The City (TBTC), which are not in RTR (See Lima 2019, 2021). As a consequence, RTR has little representation from minority groups, such as migrants, black people and LGBT + groups who, in turn, have been more connected to IHN and TBTC. People outside the coalition do not act to thwart RTR’s coalition building. IHN and TBTC have participated in RTR protest rallies, but this type of once-off partnership has not taken place often. Negotiating this difference means to articulate a routine set of actions with various groups in the coalition that are acceptable to most of the members. In the end, the majority of participants agreed that institutional political action is the main RTR tactical approach and, despite internal dissent, the group has found common ground on which to act.

Opinions within the RTR coalition differed, for example, as to whether to push for rent certainty or rent control. In this context, rent certainty refers to rent being revised periodically, and tenants know how much they will pay, whereas with rent controls the state defines rent prices in order to control the rental levels and make it affordable. Several RTR participants mentioned that certain groups, such as tenants, were more in favor of rent control, as they argued rent certainty was insufficient to make housing more affordable. Unions and center-left parties were in favor of rent certainty. A possible explanation for these two different views within the coalition might be that institutional actors supported rent certainties because it was a more moderate approach and more likely to be achieved than rent controls. But as noted by participants, this discussion subsided after some meetings and the coalition agreed on rent controls. According to this coalition participant, a representative of a leftist party: “So a point came in the crisis where everybody has recognized that the crisis has come so deep now that the demands now are very clear to everybody that there is no room for private market intervention” (Participant 24). Other participants agree that the worsening of the housing crisis made them realize that rent control was not too far-reaching and that the current rent stabilization measures—Rent Pressure Zones—are not sufficient to keep rental prices affordable. So it seems that it was both deterioration of circumstances and the need to maintain some type of unity among different views in the group sustained the movement around this particular point. Members’ commitment to fair rent and security of tenure played a critical role in maintaining cohesion and connection between the coalition and their demands. The coalition now supports the implementation of a rent freeze in tandem with security of tenure (RTR 2020).

MEL faces similar challenges. For example, some of its members reject the participation of political parties in the coalition. The solution found was that MEL generally avoids party politics. In practice, it means the members are free to join political parties, but party-related activities cannot be associated with MEL. In relation to disagreements when it came to the policy demands of the groups, I did not find any. MEL members mentioned internal pressure for another open letter, arguing the need for expanding the movement even more, while others mentioned the group needs more internal structuring, but so far it is the original letter published in 2017 that still works as the main aggregator among the various stakeholders. When asked about actions outside the institutional space, most MEL participants see direct action (i.e. building occupations and eviction disruption) as complementary to their own actions, but they do not employ it.

Thus, in contrast to RTR, some MEL members implement direct action independently. An example of this is that one of MEL’s most distinctive and active members, Habita Association, has staged numerous protests and occupations and has prevented illegal evictions. Moreover, MEL’s members see Habita’s actions as complementary to their own, as suggested by this activist member of a tenant association: “There is a great complementarity. If it is necessary to have occupations of houses and minister's offices with banners there is also a need for a more unitarian and broader movement capable of uniting different associations and putting pressure on political power” (Participant 32). In the same vein, an interviewee from RTR alluded to the notion of tolerance and respect for different tactics within the housing movements, declaring that “For a campaign like this to work, it has to find a way to give everybody their space, so they can do what feels right to them. They [non-institutional groups] have put efforts into it and we can combine what everybody brings to the table” (Participant 6, party representative).

In a similar way, a contingent of anarchists and other radicals participate respectfully in MEL’s activities, while more radical members prefer to stay away. For example, two interview participants from an anarchist background mentioned that they are members of Habita, but they do not engage in MEL’s activities. Ultimately, MEL’s participants have common ideological leanings although not all of them considered themselves radical leftists—or even leftists. They fit comfortably under the broader anti-housing financialization umbrella, and include migrants, and black and Roma people, such as in the case of the Habita Association.

When talking about overcoming these challenges, both coalitions adopt a general principle of encouraging identity preservation and respect for diverse tactics, with special attention to avoiding exposing the group’s internal dissent in public. To the interviewees, a commitment to the coalition and respectful interactions matter when it comes to the sustainability of the coalition and contribute to its longevity. As one of the respondents from RTR noted,

The biggest challenge is getting everyone to agree, and that is why I think that let people go and do the wrong thing, go and do things that I think are crazy. Go and do it. Will I criticize them in public? No, I won’t. If they do something really outrageous, I would say ‘I don’t agree with that’, but so far Raise the Roof has managed to achieve some internal balance (Participant 3).

On an analogous note, a member of MEL summarized their way to achieve a positive organizational interaction, to manage cooperation and to avoid conflict, as explained by this activist:

“There are three principles: independence, balance and collective work. The balance is not to invest in conflicts. All organizations can speak for themselves; MEL has no control. MEL is a space of communication, not an institution” (Participant 33).

To overcome the challenges of collaboration, coalition groups turn to their capacity to interact and employ what the literature has called “bridge builders,” which are usually those members with multiple memberships in different groups, who encourage interaction and negotiate among members. For example, interviewees 3, 13, 42 and 44 were members of at least three different organizations, from homeless rights, to workers’ and women’s issues. Next, I consider how key attributes of coalition building and maintenance influence the outcomes of the two coalitions.

### Coalition outcomes

The form of coalitions and how they deal with conflict play an important role in coalition outcomes. Favorable policy outcomes are also associated with their ability to achieve desired outcomes. This ability, in turn, can assist as an indicator of a coalition's ability to effectively achieve concrete goals. Coalition outcomes in this study are specified as 1) signs of a successful or effective coalition and 2) whether the coalition accomplishes its goals.

Despite the difficulties in finding common ground and coordinating actions, the RTR and MEL coalitions did succeed in putting together a number of activities, culminating in alliances that are lasting longer than previous attempts. The factors influencing coalition outcomes include its form and goals, as well as the nature of the coalition's target. Here I consider the extent of the coalitions’ capacity to organize members to achieve specific goals, such as staging a demonstration, getting mass media visibility, pooling resources, and coordinating efforts (Staggenborg 2015b). The structure of MEL, with its less formal membership mixing individuals and entities, is important in allowing organizing to be accomplished by joint integrated action. This broad coalition of academics, tenant unions and activists as well as other actors, made it possible to unleash a city‐wide campaign. A significant outcome of these developments is the way in which atomized and diverse acts of collective resistance have contributed to building a movement. Some interviewees observed that MEL is about creating a movement that turns into a coalition: “We then want this associative movement, to create this collective and try to involve people. And in MEL we have achieved this” (Participant 40), while others felt that it was more about the aim “to put housing on the political agenda. And we did it” (Participant 37). Along with a similar ideology, social ties and unified goals, MEL was efficient in creating a mobilizing structure that enabled members to organize demonstrations and gain good visibility in the media. The group, however, seems to have less success in impacting new policies. Some members reported that MEL has gained more access to the politicians in charge of urban policies in Lisbon but, so far, their influence has been more limited. Reflecting on the success of the movement, this participant observed:

What happened was that MEL placed the housing problem more clearly in the political agenda. This fundamental merit of MEL… to bring the housing problem to the public eye… much more than concrete legislative aspects, we have not been very successful in that yet (Participant 32).

RTR has also achieved success in better defining and framing housing problems, as the issue is now more clearly established in the public agenda and it has gained media visibility. However, the presence of political actors and political parties in the coalition is an important difference between MEL and RTR outcomes. While MEL has individual political actors with membership in leftist parties (i.e. Bloco de Esquerda, Partido Comunista Português—PCP), RTR has both political actors and a strong political party presence (Sinn Féin, the Green Party, the Labour Party, People Before Profit, Solidarity, Independents4Change, and the Social Democrats). These parties proposed the motion that was later voted on in the Irish parliament. Several studies have shown that coalitions that include political parties may be more successful (van Dyke and Amos 2017; Almeida 2010). A significant result achieved by the presence of the parties that was consistently highlighted by respondents when asked about outcomes, is the passing of the multi-party motion on the right to housing. As one of the participants from a homeless charity noted:

I think we shocked ourselves in some respects in what we achieved: (a) the turnout [in demonstrations], (b) the media impact was massive, it was phenomenal in social media too. The 6pm news was dominated by it, the 9pm news too. And (c) the motion was passed. The bill, it did not pass in the end, but it showed everyone we were capable, we were close (Participant 25).

In relation to mobilization outcomes, both coalitions have experienced success in defining the depth and extension of the housing problem and have gained prominence as political forces by raising awareness and proposing policy changes. RTR has brought legislation to parliament and has also pushed most of the political parties to adopt more rightful positions on the housing issue. While effective in pushing for reforms in housing legislation and in bringing together a wide range of organizations, RTR has been less effective in achieving reforms in housing legislation and in building a mass housing movement. MEL seems to be less successful in legislative terms as well. Yet, MEL was able to create a more cohesive housing movement than RTR. MEL has also been more effective in building a movement with a high level of cohesion and has been able to expose the vulnerabilities in the Portuguese housing system. Hence, it is possible to say these two case studies have produced favorable outcomes.

## Final remarks

This study analyzed the relationship between outcomes and the mechanisms which sustain coalitions. The paired comparison of Dublin and Lisbon offers important insights into how two major contemporary housing coalitions overcame challenges, how they interacted and how they cooperated to sustain alliances. In addition, the in-depth analysis of the case studies adds to our understanding on the features of coalitions in terms of their differences and similarities. The more varied the groups in the coalition, the harder it is to create consensus. While there are many challenges in coalition building, the coalitions analyzed have been able to stimulate cross-cutting cleavage participation. Their broad representation has translated into both legitimacy and political clout for the coalitions.

Social movements rely on coalitions to mobilize the large numbers of people necessary for success (van Dyke and Amos 2017). Nevertheless, it is also well known that such collaborations are hard to create and maintain due to ideological disputes, tactical disagreements and the lack of previous ties. In the case studies, the creation of an interorganizational environment was spurred by a specific political threat—the worsening of housing conditions in both Dublin and Lisbon. This was a powerful incentive for collective action, as groups started negotiating around political differences. An important dynamic for the formation of the coalitions was that both organizations employed good political timing, as their experienced members recognized the opening for political pressure and media visibility, coupled with the cooperation of “bridge builders” and a unified narrative against the housing crisis. This process of connection and interaction are crucial to coalition formation (van Dyke and Amos 2017) showing that conducive organizational structures are key to coalition formation.

With diverse membership and a wide range of interests in support of a common political agenda, the coalitions faced challenges. Maintaining coalitions with such a variety of actions is one of the main challenges for coalition sustainability and longevity. RTR and MEL members were, nonetheless, capable of overcoming most of those challenges. Even if they are not perfectly cohesive and though they face internal frictions around tactical decisions—since some organizations pay a higher cost than others if the coalition fails—the coalitions are active in many ways. The research found that a broad ideology coupled with respect for political differences has helped overcome differences and allowed coalition members to sustain their political identities and find ways to collaborate, as also shown by Enriquez (2014) and Staggenborg (1986).

Housing mobilization in Dublin and Lisbon provides an interesting comparison for analyzing the links between the mechanisms and outcomes that sustain coalitions. More importantly, the study has shown that coalitions increase their capacity to collaborate and sustain their coalition agreement by implementing tactical approaches which allow for group members to keep their identities and dissipate dissent through a tolerance for differences. In relation to outcomes, it has been found that the housing coalitions were successful in attracting visibility to the housing crisis, but so far, their policy results have been somewhat limited. The analysis has shown that more structured coalitions that include political parties as institutional allies have been more successful in pushing for reforms in housing legislation, but they lack inclusivity when it comes to minority groups. In Dublin, minority groups tend to organize outside RTR, in more horizontal and loosely organized housing groups, such as the IHN. These groups have been unable to find a common approach, which has led to fragmentation rather than cooperation among housing movements (Lima 2021). In Lisbon, MEL and its associated members have helped to put the right to housing on the public and political agenda (Mendes 2020). Being more concerned with the inclusion of vulnerable groups and minorities than RTR, MEL’s less formal structure has produced better outcomes in relation to the mobilization of the most vulnerable groups. These two study cases were relevant in order to explore how the structure and form of coalition activism affects outcomes. While both MEL and RTR were successful in placing the housing crisis in the public eye, turning it into a political issue, concrete policy changes are still difficult to achieve.

This thumbnail comparison of housing coalitions contributes to the literature on social movement coalitions and housing studies by analyzing the internal dynamics of cooperation in coalitions and the outcomes of social movement collaborations. In the particular field of housing, coalitions have informed and educated people about the origins of the housing crisis and denounced the impacts of this crisis. This article was written before the global Covid-19 outbreak, and since then the housing coalitions analyzed here are likely to have changed and achieved different results, such as the approval of emergency legislation to ban evictions and foreclosure during the Covid-19 pandemic. Further studies might well be conducted in order to revisit these coalitions and see if they still exist and/or expand after the pandemic; and if they do not, it is important then to understand what has led to their demise.

## Footnotes

1 Open letter MEL: https://www.peticao.online/signatures.php?tunnus=moraremlisboa&page_number=10&num_rows=10

2 Transcription from the Oireachtas regarding the Housing Motion: https://www.oireachtas.ie/en/debates/debate/dail/2018-10-03/31/

3 Until mid-2017, housing was part of the portfolio of the Minister for the Environment and Climate Action. In August 2017, a new Secretary of State for Housing was nominated, under the portfolio of the newly created Ministry of Infrastructure and Housing.
